# Characterization of *JsWOX1* and *JsWOX4* during Callus and Root Induction in the Shrub Species *Jasminum sambac*

**DOI:** 10.3390/plants8040079

**Published:** 2019-03-29

**Authors:** Ying Lu, Zhuoyi Liu, Meiling Lyu, Yuan Yuan, Binghua Wu

**Affiliations:** Fujian Provincial Key Laboratory of Plant Functional Biology, College of Horticulture, Fujian A & F University, Fuzhou 350002, China; bying_lu@sina.com (Y.L.); zhuoyi_liu@126.com (Z.L.); mllv2009@126.com (M.L.); yuanyuan@fafu.edu.cn (Y.Y.)

**Keywords:** callus, *de novo* organogenesis, WUSCHEL-related homeobox (WOX) transcription factors, *Jasminum sambac*, transgenic

## Abstract

Plant regeneration *in vitro* and the underlying molecular regulatory network are of great interest to developmental biology, and have potential applications in agriculture and biotechnology. Cell growth and re-differentiation during *de novo* organogenesis require the activation and reprogramming of stem cells within the stem cell niche of the tissues. The WUSCHEL-related homeobox (WOX) factors play important roles in the maintenance and regulation of plant stem cells and are involved in many developmental processes. However, in woody species such as the *Jasminum sambac,* little is known about the involvement of *WOX* genes in *de novo* organogenesis. Here we show that two *WOXs*, *JsWOX4* and *JsWOX1*, are implicated in callus proliferation and root regeneration, respectively. The expression of both, together with another member *JsWOX13*, are upregulated during later stage of callus formation. The *JsWOX4* is associated with callus proliferation, or cell division during the redifferentiation. The overexpression of this gene results in up-regulation of *JsWOX13* and another homeobox gene. The *JsWOX1* plays a role in root primordium initiation, as its overexpression leads to more rooty calli and more roots per callus. *JsWOX1* also possibly acts upstream of *JsWOX4* and *JsWOX13* transcriptionally. Our results provide further evidence regarding the functions of *WOX* genes in organogenesis in a woody plant.

## 1. Introduction

Plants can regenerate parts, or their entire body from a few somatic cells, an ability described as totipotency, which has great applications in agriculture and biotechnology [1,2]. Under *in vitro* tissue cultures, regeneration might take the route of somatic embryogenesis, or of *de novo* organogenesis depending on the induction conditions, plant species and the nature of explants, among others [3]. In most cases, indirect regeneration, either embryogenesis or organogenesis, undergoes a two-step procedure that requires the first induction and proliferation of callus (unorganized cell mass) from stem cell lineages within various explants [4]. This initiation step resembles the root development pathway at the molecular and cellular levels, regardless of the origin of the explants [5]. The next step of the regeneration of shoots and roots - or even of an embryo-like structure - depends on the activity of stem cells at the stem cell niche to establish apical meristem primordium, a process which is stimulated and regulated by a number of specific regulators [6,7,8,9].

In addition to residing in the layers of shoot apical meristem (SAM) and root apical meristem (RAM), stem cells in adult plants are distributed throughout the whole body along the vasculature. The known examples are pericycle cells adjacent to the xylem poles in roots or hypocotyls and the (pro)cambium cells of stems [10]. These pluripotent cells enable the plant to, post-embryonically, control the proliferation, self-renewal and differentiation of specific tissues and organs in accordance with environmental and developmental signals [10]. With this respect, the plant specific WUSCHEL-RELATED HOMEOBOX (WOX) transcription factor family, named after the founding member of *Arabidopsis* stem cell regulator WUSCHEL (WUS), plays key roles in stem cell maintenance and activation underlining diverse developmental processes and physiological events [11]. Phylogenic alignment of WOX proteins divides the family into three groups; namely the ancient clade (WOX13-like), the intermediate clade of WOX8/9/11/12 and the WUS clade with WUS and WOX1 through WOX7 [12,13]. WUS is the master regulator of the stem cell niche in the shoot meristem, where its expression is limited to cells of the organizing center (OC) beneath the stem cells [14,15]. Together with a specific signaling peptide CLV3, WUS forms a dynamic feedback loop to control stem cell homeostasis [16,17]. WUS also regulates the floral meristem initiation and the development of female and male organs [18,19,20,21]. Other members of the *WUS* clade can more or less substitute for *WUS* functions in shoot and floral stem cell maintenance [11], except the *WOX4* which has important regulation role in cambium stem cells [22,23,24]. Members of both the ancient clade of *WOX13*-like and the intermediate clade of *WOX9*-like do not have the function for stem cell maintenance [11] but are required in various developmental processes such as embryogenesis and shoot development [25,26], root development and floral transition [27]. The ancient clade and the intermediate clade either lack the WUS-box or possess a modified WUS-box [13].

Despite major advances in recent years regarding the functions and mechanism of *WOX* genes in plant *de novo* regeneration using *Arabidopsis* and other herbaceous species [9], less studies are reported in woody plants [22]. *Jasminum sambac* (L.) Ait. is an evergreen shrub widely cultivated as an ornamental, and its flowers provide important raw materials for scented tea and essential oils. In the present study, we identified and cloned four *WOX* related genes and analyzed their expression in an established callus culture and transformation system. We showed that *JsWOX4*, *JsWOX13* and a specific homeobox gene were involved in callus induction and proliferation. Whereas *JsWOX1* would function in root primordial initiation and differentiation. Our results provide a first line of evidence of how *WOX* genes might be involved in the *in vitro* regeneration in this woody plant. 

## 2. Results

### 2.1. Establishment of J. sambac Callus Culture and the Role of Auxin and Cytokinin

**Figure 1:**
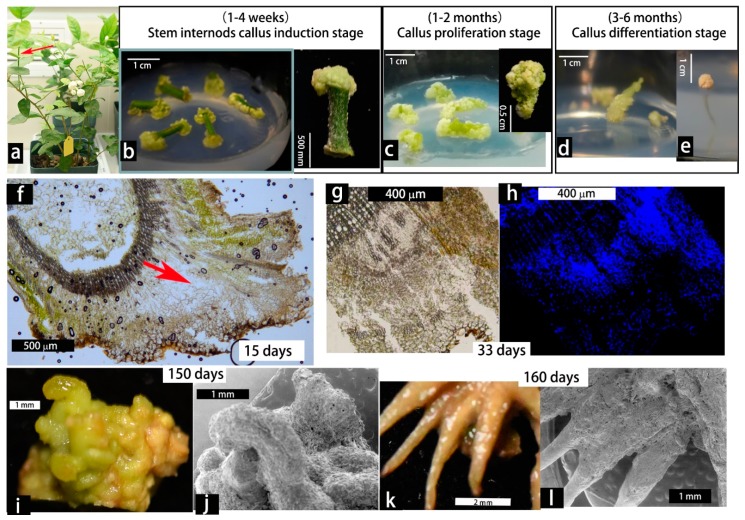
Callus induction and differentiation from young stem explants of *Jasminum sambac*. (**a**) Young stem cuttings from potted three-years-old plants grown in climate room were used as explants. (**b**–**e**) Callus induction, proliferation and morphogenesis on WPM basal medium supplemented with 4.03 mM NAA + 9.99 mM BA, refreshing every 20 days. (**f**) A cross section view of an early callus. (**g**–**h**) Sections from the same callus, one stained with DAPI. (**i**–**l**) Light microscopy and scanning electron microscopy view of granulated callus (**i**,**j**) and regenerated roots (**k**,**l**).

Successful callus culture and plantlet regeneration from the genus *Jasminum* is seldom reported in the literature. In a previous experiment, we used leaf explants from *J. sambac* for *in vitro* culture, but the callus grew very slow and eventually became brown (data not shown). In contrast, using young stem cuttings as explants (Figure 1a), callus can be easily induced and maintained under conditions tested (Figure 1a–e, Table 1 and Figure S1). The stem explants produced calli with variable efficiency in response to a wide range of combination of 1-Naphthaleneacetic acid (NAA, from 0 to 112.78 mM) and 6-Benzylaminopurine (BA, from 0 to 93.25 mM) supplemented to the WPM basal medium (Table 1 and Figure S1). No difference was found when MS medium was used instead of WPM (data not shown). 

Although callus induction from stem explants could be initiated more or less in all combinations of NAA and BA, auxin is better than cytokinin when used alone (Table 1). In terms of quantity and quality of the formed calli, lower auxin to cytokinin ratio (from 11:89 to 50:50) achieved better results for callus maintenance. It seemed that the proportion between the added auxin and cytokinin - other than their concentration - was more relevant for callus induction, however, optimal efficiency was found when the added total amount of NAA + BA ranges from 7.02 to 48.34 mM (Treatment 1 through 17 in Table 1). Particularly, the combinations of 1.07 mM NAA + 8.88 mM BA (Treatment 7), 8.06 mM NAA + 6.66 mM BA (Treatment 11) and 4.03 mM NAA + 9.99 mM BA (Treatment 12) showed the highest callus-inducting efficiency from stem explants in the first month of culture (Table 1 and Figure S1). In prolonged culture on these three media, adventitious root regeneration were seen in a few callus (Treatments 12, 11 and 7), while in other treatments most of the calli only expanded in mass but without any organogenesis. These calli eventually turned brown and died out (Table 1 and Figure S1). Although it is well known in *Arabidopsis* or some other plants that cytokinin-rich medium can induce shoot regeneration from callus [28]. This simple rule seems invalid in the case of J. sambac under the tested conditions (in this case NAA and BA), or it would otherwise be necessary to reexamine with more phytohormone combinations or with medium-turning.

In the subsequence experiments, we used WPM basal medium supplemented with 4.03 mM NAA + 9.99 mM BA (Treatment 12) or 1.07 mM NAA + 8.88 mM BA (Treatment 7) for callus induction, proliferation and organogenesis study. These two treatments obtained similar results but the former gave rise to more root-regeneration. Under these culture conditions, calli of yellow-green to pale green was induced within 1–4 weeks (Figure 1a,b). During sub-culture within 1–2 months, the callus continued to grow and mostly remained undifferentiated, with granulated cell clamps on the surface (Figure 1c). Occasionally budding-like structures were observed, but they turned brown after one month and did not give rise to stem regeneration (Figure 1d). Only adventitious root-like structures were induced and further developed (lower than 30% of calli) during the extended culture stage of 3–6 months post explanting (Figure 1e). 

The callus founder cells were originated from procambium or cambium, as they resembled dividing parenchyma cells (Figure 1f). By using DAPI staining it could be shown that DNA-rich cells in callus marked the proliferating region (Figure 1g,h). In a prolonged period of culture, the surfaces of calli displayed some compact and friable bud-like structures that were incapable of shoot regeneration, while roots induction was initiated in some calli (called rooty callus) (Figure 1i–l). Thus, in view of *de novo* shoot-regeneration competency, *J. sambac* is among those of "recalcitrant" plant species, which awaits further turning of the culture conditions.

### 2.2. Analysis of WOX Gene Expression during Callus Induction

**Figure 2:**
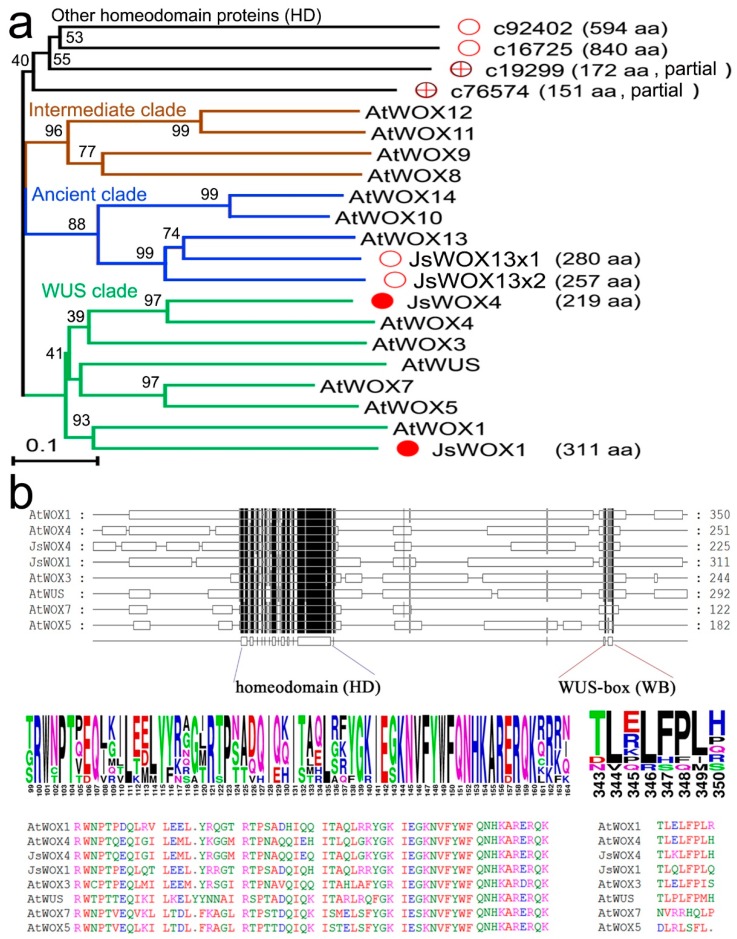
(**a**) A phylogenetic tree of the cloned WOX-related homeobox proteins from *J. sambac* together with *Arabidopsis* ones showing the three clades of the plant WUS/WOX family. (**b**) Amino acid sequence alignment showing protein-domain structure of the WUS clade and, the conserved sequences of both the homeodomain and the WUS motif. Sequence alignment was conducted using MUSCLE (https://www.ebi.ac.uk/Tools/msa/muscle/) and the Neighbor-Joining tree was constructed using MEGA X (https://www.megasoftware.net/) with a bootstrap number 1000. The full-length sequences of *J. sambac* WOXs can be found in the Supplementary File.

Regeneration competency, or totipotency, relies on transcriptional reprogramming in the stem cell niches in response to external and internal cues [2,4,9]. Given the importance of *WUS/WOX* genes in regulating stem cell activities, we first analyzed the expression of *WOX*-like genes during callus induction and differentiation of *J. sambac*. Because of the lack of genomic sequence, we identified unigenes by BLAST search from our previous transcriptome database generated from a mixed RNA sample of flower, stem and leaves tissues of *J. sambac*. Using gene-specific primers, we successfully cloned four full-length *WOX*-like cDNAs, designated as *JsWOX1*, *JsWOX4*, *JsWOX13x1* and *JsWOX13x2* based on their similarity in amino acid sequences with respective *Arabidopsis* orthologs (Figure 2a). Another four ESTs, assigned as *c92402*, *C16725*, *C19299*, *C76574*, were also identified as related homeobox-containing homologs (HD) (Figure 2a). Alignment of amino acid sequence reveals the conserved WUS signature motif in addition to the homeodomain in the *JsWOX1* and *JsWOX4*, which places them into the WUS clade (Figure 2b). The other two cDNA, *JsWOX13x1* and *JsWOX13x2* seem to be transcript variants of a same gene. The full-length sequences are listed in the Supplementary File.

**Figure 3:**
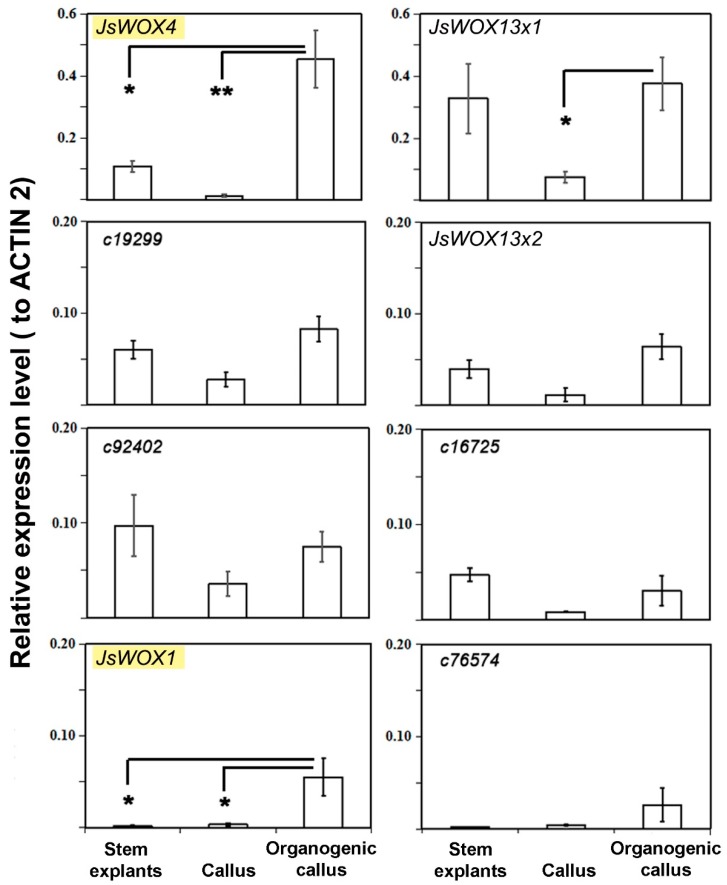
Relative expression of *WOX* and homeobox genes during callus culture of *J. sambac* using *ACTIN2* as an internal reference. Error bars represented SE of three biological replications, which were pools of five to ten callus (Student’s test, *<0.05, **<0.01).

By quantitative reverse transcriptase PCR (qRT-PCR) we could show that in the three stages (stem explants, two-month proliferating callus, three-month differentiating callus), only *JsWOX1* and *JsWOX4* were upregulated in the latest stage, whereas the other genes were not significantly altered (Figure 3). The expression of *JsWOX13x1* displayed variation in the callus induction phase (Figure 3). The detected transcripts of *JsWOX1* were relatively low in both stem explants and in callus proliferating phases. Its expression increased by more than ten folds in the later stage when granulated or when rooty callus appeared. The *JsWOX4* was the highest expressed gene in the latest stage with an average value of 0.45 (2^-DDCt^ relative to *ACTIN2*). 

### 2.3. In Situ Detection of JsWOX1 and JsWOX4 Transcripts in Calli

**Figure 4:**
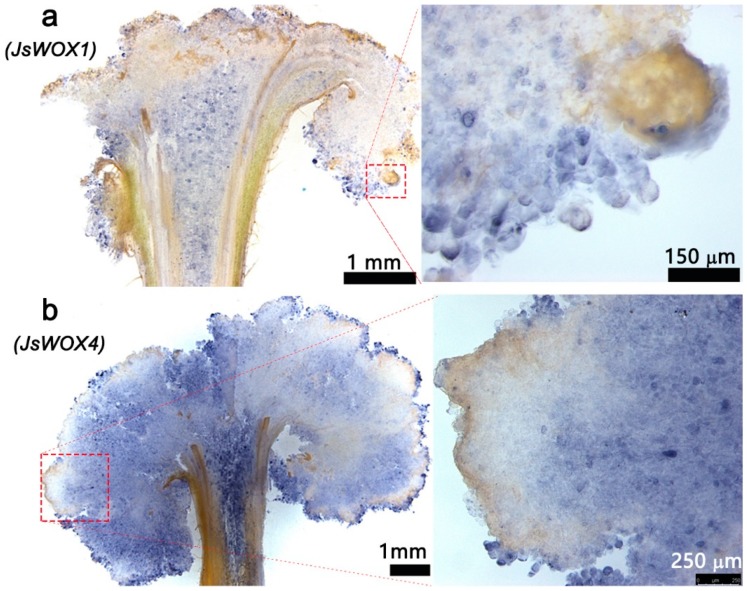
*In situ* hybridization of *JsWOX1* and *JsWOX4* transcripts using digoxigenin-labeled antisense fragments as probes on cryo-sections (40–50 mm thick) from four-months-cultured callus.

Since qRT-PCR data does not reveal cell-specific information which is important for deciphering stem cell activities, we further conducted *in situ* mRNA hybridization in callus sections using digoxigenin-labeled antisense fragments of *JsWOX1* and *JsWOX4*, respectively. In the later stages of callus formation, the mRNA hybridization signal was stronger for *JsWOX4* than *JsWOX1*. The *JsWOX1* was found to express both in a few marginal cells and in the central parenchyma cells. The identity of such cells was difficult to justify, but seemed likely to associate with root primordium initiation (Figure 4a). The mRNAs of *JsWOX4* were more widely detected in cambium or procambium cells from the stem explants, and also in most callus cells (Figure 4b). This is similar to that reported in *Arabidopsis* [23,24,29] and Populus [22], where the *WOX4* orthologs have been demonstrated to be exclusively expressed in phloem cambium stem cells and plays important roles for cambium cell proliferation. Therefore, it could be possible that the mass of the callus is likely derived from cell division of the cambium or procambium progenitor cells.

### 2.4. Effects of Ectopic Expression of JsWOX1 and JsWOX4 on Callus Morphogenesis and Gene Expressions

**Figure 5:**
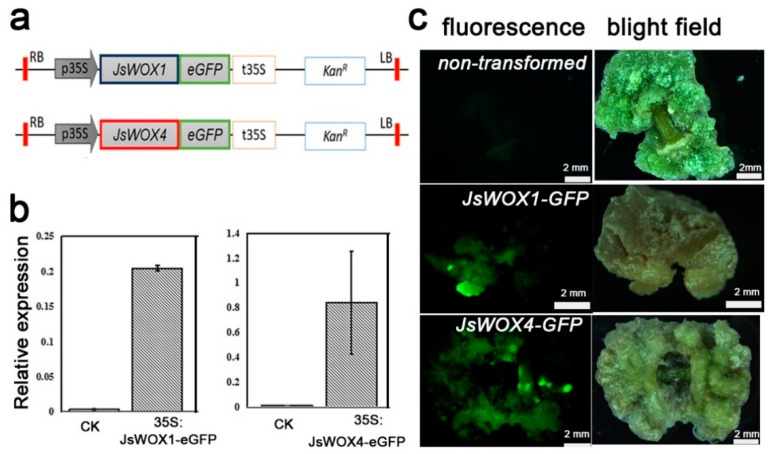
Construction of transgenic calli for overexpression of *JsWOX1* and *JsWOX4*. (**a**) The binary cassettes for ectopic expressions of GFP fusions with JsWOX1 or JsWOX4. (**b**) Quantitative RT-PCR of *JsWOX1* and *JsWOX4* transcripts performed at two months post transformation. Non-transformed calli were used as controls (CK). Error bars represented SE of three biological replications, which were pools of five to eight callus. (**c**) Fluorescence stereo microscopy images of calli at one month after transformation.

Having shown that the cell-specific expression pattern of *JsWOX4* marked the proliferating callus cells and that the *JsWOX1* seemed to express only in tips of the callus, probably at the primordial niche, we next produced transgenic calli of the respective full-length cDNA in fusion with a *GFP* (Figure 5a), using calli of one month as recipients for *Agrobacterium*-mediated transformation. After two months in selection medium the transformed calli showed an increase in transcript accumulations of *JsWOX1* and *JsWOX4*, up to approximately 20 and 80 folds, respectively (Figure 5b). The fluorescence signals from the fusion GFP were examined during the processes (Figure 5c). Although the transgenes were driven by the constitutive CaMV (Cauliflower Mosaic Virus) 35S Promoter, the GFP signals of JsWOX1- or JsWOX4-fusion was somehow consistent with the respective *JsWOX1* or *JsWOX4* expression pattern by *in situ* hybridization as shown above, meaning that the distribution of the two WOX proteins may also regulate at the post transcriptional level. Of course, possibilities of heterogenic transformed cells could not be excluded here.

**Figure 6:**
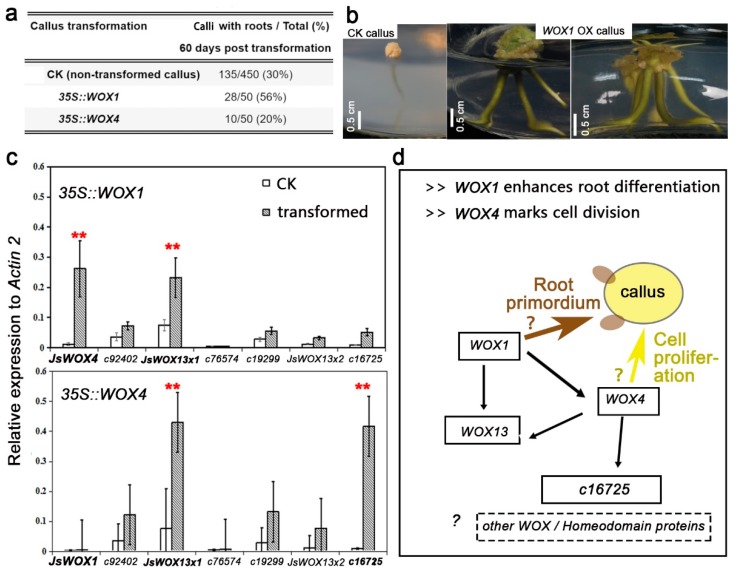
The effects of ectopic expression of *JsWOX1* and *JsWOX4* on callus morphology and gene expressions. (**a**) Comparison of callus rooting at 60 days post transgenic. Transformation repeated two times with merged results shown. The non-transformation data represent sum of ten replications. (**b**) Representative images of calli showing an enhanced effect of root differentiation by overexpressing *JsWOX1*. (**c**) Quantitative RT-PCR determination of transcript abundance of *WOX* and related homeobox genes in transgenic and control calli at 60 days post transformation. Error bars indicated SE of three biological replications, which were pools of five to six calli. Data represents mean for three biological replications with Student’s t-test: * < 0.05, ** < 0.01. (**d**) Schematic view of suggestive roles of *JsWOX1* and *JsWOX4* in callus induction and differentiation. *JsWOX4* appears to be involved in callus proliferation or cell division, it also seems to be upstream of *JsWOX13* and of at least another homeobox gene. The JsWOX1 might have a promotive role in root primordium differentiation and could activate gene expression of *JsWOX4* and *JsWOX13*.

Morphologically, overexpression of *JsWOX1-GFP* could lead to the increase of root-generating calli (rooty calli) by nearly two times, whereas *JsWOX4-GFP* did not change the regeneration status or even inhibited slightly root-forming in the transgenic calli (Figure 6a, no significant test). It was also observed that most of the calli overexpressing *JsWOX1* possessed more number of roots per callus as compared with others, suggesting an enhanced root primordial differentiation (Figure 6b). These induced root-like structures looked much thicker in appearance.

qRT-PCR analysis revealed that both *JsWOX4* and *JsWOX13x1* were significantly upregulated in *JsWOX1* overexpressing calli, while *JsWOX13x1* and a homeobox gene (*c16725*) were activated in transgenic calli of *JsWOX4* (Figure 6c). These results suggest the existence of a multifaceted genetic interaction between the WOX transcription factors in callus induction, proliferation and root regeneration.

## 3. Discussion

*De novo* organogenesis in plants depends on stem cell activities under favored conditions. Understanding the molecular mechanism underlying the regeneration processes is of central importance for plant developmental biology, and has potential application in agriculture [9]. Our knowledge on the molecular regulation of pluripotency and regeneration pathways are largely gained from systematic studies in model plants such as *Arabidopsis* [9,13]. However, regeneration capacity, hence the genetic mechanism, of the same kind of explants can vary among different species and even in different genotypes of the same specie [2]. *J. sambac* is among the woody species with less information available despite its horticultural importance. It is also considered to be ‘recalcitrant’ in terms of *de novo* shoot regeneration *in vitro*, to our knowledge. In this paper, we have identified two WUS-related homeobox transcription factors which play putative roles during callus induction, proliferation and root regeneration, in an established *in vitro* system using stem explants from *J. sambac* (Figure 6d). 

The *JsWOX4*, which is expressed during later stage of callus expansion and in cells of (pro)cambium origin (Figure 3 and Figure 4), seems to be involved in cell division during tissue re-differentiation. The *Arabidopsis WOX4* is required for both auxin-dependent cambium proliferation and extended root and stem thickening functions, in downstream of a specific leucine-rich repeat receptor-like kinase (PXY) [23,24,29]. This WOX4-centered CLE-RLK-WOX cascade has also been unveiled in Populus trees [22]. Thus, it would be interesting to further examine the exact functions of *JsWOX4* in *J. sambac.* Moreover, overexpression of *JsWOX4* leads to the up-regulation of the *JsWOX13* (Figure 6c), a member of the ancient clade. Its *Arabidopsis* ortholog plays a role in root and floral development [30]. The closely related rice homolog, *OsWOX13* is involved in drought resistance and early flowering [31]. However, the signaling mechanism involving *JsWOX4* and *JsWOX13* is still not known yet.

Furthermore, our data suggests that the *JsWOX1* may play an important role in root regeneration. It is expressed around the surface of granulated callus during a much later stage, and its overexpression promotes root-forming ability of the calli (Figure 3, Figure 4, Figure 5 and Figure 6). It is also interesting that *JsWOX1* can induce the expressions of *JsWOX4* and *JsWOX13x1* (Figure 6c). The closest *Arabidopsis* homologs, *AtWOX1*, has been shown to be an important regulator of lateral-specific blade outgrowth and margin-specific cell fate in leaf development [32]. Other *WOX* members implicated in root development are *AtWOX7* [33], *AtWOX11* and *AtWOX12* [34]. We postulate that *JsWOX1* might be necessary for stimulating root primordium initiation, although further evidences are needed. 

## 4. Materials and Methods

### 4.1. Plant Materials and Culture Conditions

The three-year-old plants were cultivated in pots under climate room with a light/dark cycle of 16h/8h and temperature of 26 °C/22 °C. Young shoots of these plants were cut into 0.5-cm-long stem segments and used as explants. For the basal culture medium, WPM [35] and MS [36] were used, supplemented with various concentrations of NAA and BA plus agar 6.5 g L^−1^ and sucrose 20 g L^−1^. Cultures were maintained under light intensity of 1200-2000 lux (LED light source), 23–27 °C temperature and 12h/12h light/dark. Every 20 days the cultures were transferred to fresh media. Callus induction and the subsequent organogenesis were tested with the same set of medium supplemented with various total amounts and ratios of NAA to BA, as indicated in the results. The pH of all culture media was adjusted to 5.80 with NaOH and HCl before adding the agar.

### 4.2. Transverse Sections, Histological Observation and Scanning Electron Microscopy (SEM)

For tissue section preparation, fresh stem explants and calli of various stage during induction fixed in 4% paraformaldehyde for 48 h, kept at 4 °C. The OCT embedded tissues were subjected to transverse sectioning using a microtome (Leica CM1950, Leica Microsystems, Germany) set for 40–50 mm thick. Fluorescence microscopes under a stereoscopic Leica M205 FA, or an inverted Leica dmi8, were used for histological examination. For SEM analysis, Hitachi TM3030Plus was employed according to the protocol from the manufacture. Analysis was conducted on five to ten samples.

### 4.3. Full-Length cDNA Cloning and Agrobacterium-Mediated Transformation

We identified several EST homologs in our previous transcriptome database generated from a mix RNA sample isolated from leaves, young stems and flowers, using software BioEdit’s local BLAST search with sequences of the *AtWUS* and other plant *WOXs*, setting a cut-off E-value of −5. After manually curated sequence analysis, we assigned *JsWOX1*, *JsWOX4*, *JsWOX13x1* and its variant *JsWOX13x2* on the four *WOX* genes. These were full-length cDNA sequences, in addition to four related homeobox genes. Accordantly, gene specific oligonucleotide primers were designed to amplify the full-length CDS sequences. The primer pair for *JsWOX4* was: caccAGATCTATGGTAGTTGAAGCCACCATGAAGGTTC, and AGATCTTCGTCCTTCGGGGTGCAAGGGAAAGAG; For *JsWOX1* was: caccAGATCTATGTGGATGATGGGATACAACGATGGAGG, and AGATCTGTTCTTCAATGGAAGGAACTCAAAAAACTGATAAG. The full-length CDSs were then used for cloning into the pENTR™/D-TOPO® (Thermo Fisher Scientific, Shanghai, China) and subsequently for subcloning into binary vector pK7FWG2 [37], respectively.

Transformation of calli was conducted using *Agrobacterium* stain GV3103. A single colony of the recombinant *Agrobacterium* was grown in liquid LB medium at 28 °C with shaking at 220 rpm to an OD_600_ of 0.5. The harvested *Agrobacterium* suspensions with an adjusted OD_600_ of 0.6 in ½ MS medium containing 100 mM acetosyringone were used for incubation of seven-days precultured stem explants. After 20 min incubation, the bacterial suspensions were removal and the inoculated explants were co-cultivated at 20 °C for five days in the dark in WPM medium supplemented with 2 mg/L 6-BA, 0.2 mg/LNAA and 15 mg/L acetosyringone. Subsequently, these co-cultured explants were rinsed five times with sterile water, and twice with 180 mg/L cefotaxime solution. The treated explants were cultured in medium supplemented with antibiotics (180 mg/L cefotaxime and 220 mg/L kanamycin) and the culture media was refreshed every 20 days. 

For GFP epi-fluorescence detection, calli of two months post transformation in culture were sampled and examined under a fluorescence microscope (Leica M205 FA) setting the excitation wavelength filter for GFP at 450–490 nm.

### 4.4. In situ mRNA Hybridization

The digoxigenin-labeled antisense probes contained nucleotides 435 to 621 bp and 292 to 491 bp downstream of the start codon for *JsWOX1* and *JsWOX4,* respectively. They were generated using the DIG RNA Labeling Kit (SP6/T7) from Roche (Germany) with full-length cDNA as templates according to protocol provided by the kit. Calli of three month culture were fixed and sectioned as before and used for hybridization according to the kit manual (DIG High Prime DNA Labeling and Detection Starter Kit II, TransGen, Shanghai, China). Images were taken with a Leica DMi8 inverted microscope. Observation with five to ten replications was carried out.

### 4.5. Quantitative RT-PCR

Total RNA was isolated from stem explants and calli of various stage of culture or post-transformation using an RNAprep pure Kit (TIANGEN Biotech, Beijing, China). First-strand cDNA synthesis was carried out using the TransScript One-Step gDNA Removal and cDNA Synthesis SuperMix (TransGen, Shanghai, China). Quantitative PCR was performed on a Roche LightCycler 96. The relative expression level of target genes were calculated by the 2 (-DeltaDeltaC(T)) method [38]. Each determination contained three technical repeats. We pooled five to ten explants or calli as one biological replication and quantification of RT-PCR were normally conducted with three biological replications. The oligonucleotide primers used for qRT-PCR are listed in Table 2.

### 4.6. Sequence Alignment and Phylogenetic Tree

*Arabidopsis WUS/WOXs* sequences were retrieved from GenBank with the following accession number: *WUS* (At2g17950), *WOX1* (At3g18010), *WOX2* (At5g59340), *WOX3*/*PRS* (At2g28610), *WOX4* (At1g46480), *WOX5* (At3g11260), *WOX6*/*PFS* (At2g01500), *WOX7* (At5g05770), *WOX8* (At5g45980), *WOX9* (At2g33880), *WOX10* (At1g20710), *WOX11* (At3g03660), *WOX12* (At5g17810), *WOX13* (At4g35550), *WOX14* (At1g20700). Sequence alignment was carried out using MUSCLE (https://www.ebi.ac.uk/Tools/msa/muscle/), and a Neighbor-Joining tree was constructed using MEGA X (https://www.megasoftware.net/) with 1000 bootstrap number. Sequence logo images were generated by the WebLogo 3 (http://weblogo.threeplusone.com/). The full-length sequences can be found in Supplementary File.

## Figures and Tables

**Table 1:** Phytohormone effects on callus and adventitious root induction of stem explants from *J. sambac* after two months in culture.

Treatment	WPM Medium + NAA + BA (M)		Ratio NAA/BA	Percent Explant with Callus (%) (1 Month)	Percent Explant with Rooted Callus (%) (2 Months)	Extended Callus Culture (up to 6 months)
1	8.06	0	100/0	80 (*n* = 22)	0	browning, dying
2	6.04	1.67	78/22	83 (*n* = 20)	0	browning, dying
3	4.03	3.33	55/45	85 (*n* = 30)	0	browning, dying
4	2.01	5.00	29/71	65 (*n* = 18)	0	browning, dying
5	0	6.66	0/100	15 (*n* = 18)	0	browning, dying
6	2.69	8.88	23/77	86 (*n* = 400)	3	most browning
7	1.07	8.88	11/89	98 (*n* = 450)	6	pale green, stable
8	0	8.88	0/100	10 (*n* = 400)	1	browning, dying
9	16.11	0	100/0	86 (*n* = 23)	13	some browning
10	12.08	3.33	78/22	96 (*n* = 24)	12.5	browning
11	8.06	6.66	55/45	98 (*n* = 23)	13	dark green, stable
12	4.03	9.99	29/71	98 (*n* = 126)	30	pale green, stable
13	0	13.32	0/100	40 (*n* = 23)	0	browning, dying
14	48.34	0	100/0	60 (*n* = 19)	0	browning, dying
15	36.25	9.99	78/22	68 (*n* = 18)	0	browning, dying
16	24.17	19.98	55/45	85 (*n* = 25)	4	some browning
17	12.08	29.97	29/71	34 (*n* = 29)	7	some browning
18	0	39.96	0/100	10 (*n* = 23)	0	browning, dying
19	112.78	0	100/0	14 (*n* = 13)	0	browning, dying
20	84.59	23.31	78/22	15 (*n* = 18)	0	browning, dying
21	56.39	46.63	55/45	8 (*n* = 14)	0	browning, dying
22	28.20	69.94	29/71	40 (*n* = 22)	4.5	browning, dying
23	0	93.25	0/100	4 (*n* = 10)	0	browning, dying

**Table 2:** Oligonucleotide primers used for qRT-PCR.

Gene	Forward (5′-3′)	Reverse (5′-3′)
*JsWOX1*	ACACCGTCGGCTGATCAAAT	ATTCCAGTTGACGTCGCCTT
*JsWOX4*	TAGAGGAGGAATGCGAACGC	TTCTGTCTCTCGCGTGCTTT
*JsWOX13x1*	ATTACGGGCAGGCAGAGATG	CCGTGTTGGCATAACTCTGC
*JsWOX13x2*	TGTACCCTGGTGGCCATAGA	TCTGCTTGCTTGGAGTTCCA
*c92402*	TTTTCGCAGCTTGCTTCCAC	CGGGACCCAACGATGAGAAT
*c76574*	GCTCAAGTGGGCATGGGATT	GCACCTCTCTCGTATCGTGT
*c19299*	GCTGTGTCTAGGTGCATGGT	TCCGCTTCATCGTACACTCC
*c16725*	AAAGTTGTGGTGGAGTGGCA	CGACAGTCCCGAAACCAAGA
*JsActin2*	TCTCTATGGTAACATTGTCCTG	TCTCTATGGTAACATTGTCCTG

## Supplementary Materials

The following are available online at https://www.mdpi.com/2223-7747/8/4/79/s1, Figure S1: Effect of NAA/BA on callus induction, Supplementary file 1: sequences of *WOXs*.
