# Supplementary material for: Characterization of JsWOX1 and JsWOX4 during Callus and Root Induction in the Shrub Species Jasminum sambac
Source: Plants (Basel). 2019 Mar 29;8(4):79. doi: 10.3390/plants8040079 (PMC6526479; doi:10.3390/plants8040079)
Supplement: Supplementary file 1 [file plants-08-00079-s001.zip › supplementary file1-sequences of WOXs.pdf]

Amino acid sequences

>JsWOX1
MWMMGYNDGGGDFDPTGWKFRPLVPRPAATSATVNPHCFSRVNPTDLLSLNHHLCVAEKNKREFNTQQVV
ASSRWNPTPEQLQTLEELYRRGTRTPSADQIQHITAQLRRYGKIEGKNVFYWFQNHKARERQKRRRQLESVSDS
EQQPRSNMEISEKKESSDANRTIYECEQTKNWASPTNCSTIAEKTLPSTQKTPEAECKSDGWLQFDEVAHELQQ
RRSQLAEMNATWQTTKQSINPTNDVIITFLPARRDDEFQTLQLFPLQSSDGSGGGDCGDNDDHSQENVVQTS
ASGMNSDLSSYQFFEFLPLKN
>JsWOX4
MVVEATMKVHQFARGFWDQHVHETPSLTLGCKRFRPLAPKHLTAKNSDTNDPIFDLKSFIRPESGPIKIGSSDY
HKKESAQVETHPGGTRWNPTQEQIGILEMLYRGGMRTPNAQQIEQITAQLGKYGKIEGKNVFYWFQNHKARE
RQKQKRNSLGLSSHSPRTPPSILNTSLLLSENKGDEDSPHKRKCRPWTFEGFDEEKRYCTEDEDEDRTLKLFPLHP
EGR
>JsWOX13x1
MEWNKPPPQQAAHPEELNGGGGGGGNLNVNGGGMFVKVMTDEQMEVLRKQIAVYATICEQLVELHKSLTS
QHDLAGARLVNLYSDPFVTSGGHKITGRQRWTPTPVQLQILERIFDQGNGAPSKQKIKEITAELCQHGQISETNV
YNWFQNRRARSKRKQQATQQNHAESEVETEVESPNEKKTKPDDFQTPQIASSKPEDLCFQNPEANSAMHSM
DPRASKPEPMLPPEGSSHHSGNFGQMSFYGNMLVNQRMDNMIGKMEVPGNYNPYLQPDDYNMTR
>JsWOX13x2
MEWGKQPNVQQVAEELNNGGIFVKVMTDEQIEILRKQIAAYAIICEQLVHLHKSFTLQNDLPGYGNLYYGPLMY
PGGHRISARQRWTPTPVQLQILERTFDLGNGTPSKQRIKEITSELSQHGPISETNVYNWFQNRRARSKRKQQSA
PLYSAEPEADTDVESQSEKTEKPQQHVPSSRSEDNPEFSSATHSMDPFRMRKPERILQPEDSLKRNGSSGQLPY
GSMLSNPGVEHLIEKMEVPGSYSSYPQLDGFDPTG
>c19299
ESENMNWILANSKPCPKCKRPIEKHQGCMHMTCTPPCKFEFCWLCLGAWSEHGERTGGFYACNRYETAKQE
GVYDEADRRREMAKNSLERYTHYYERWASNQSSRHKALVDLHQMQSVHLEKLSEVQSQPESQMKFIIESWQ
QIVECRRVLKWTYAYGYYLPEHEHTKKQFF
>c92402
MPAMESIEAPANDDGMAGFLPLASASQQPYVSELLSFTLDRLHKEPELLRVDAERIQRQMQEVAVGNFRAFIA
AADALLEIREEVSSIDRHLESLIAEIPKLTSGCSEFLESAEQILEKRKMNQTLLTNSSTLLDLLEIPQLMDTCVRNGN
YDEALDLEAFVVKLTTMHPKIPVIQDLAAEVKQTTQSLLSQLLLKLKSNIQLPECLRIIGYLRRIGVFSEYEMRLQFL
RCRQAWLTGILDDLDQRNPYEYLKGMINCHRMHLFDVVNQYRAIFADDTSGIEENHDGGLLFNWALHQITSH
LKTLKVMLPKISEGGSLSNILDQCMYCAMSLGWVGLDFRSLLPPLFEEAVVNLFSKNMSTAVENFQLVLDSHR
WVPLPAVGFPANSLGEENQDDVTPPSNLMEHPPLAVFINGVSAAMNELRPCAPLSLKNLLAQELVKGLQAVSD
SLLRYKTTRMLRDNESLLFLKLCHAFIEVAFPHCVTCFGRCYPGGASLIADAKNLFDGINRLLENSRSRALPKQVH
RADSKSTSENGNQPVAENGNQPVAENGIVHSDEQAASDNDREKEHNNISPQRRSSVTDEDNSDPLPQEVKT
>c16725
MAMVVQQHRESSSGSGSGGGSINKHLDTGKYVRYTAEQVEALERVYSECPKPSSLRRQQLIRECPILSNIEPKQI
KVWFQNRRCREKQRKESSRLHSVNKKLSAMNKLLMEENDRLQKQVSQLVCENGYMRHQLQSATTDASGESA
STTPRNAIRDANNPAGLLSIAEETLAEFLSKATGTAVDWVQMPGMKPGPDSVGIFAISQSCGGVAARACSLVSL
EPTKIAEILKDRSSWFRDCRSVEVFTMFPAGNGGTIELLYTQTYAPTTLAPARDFWTLRYTTTLENGSLVVCERSLS
GTGAGPSAASASQFLRAEMLPSGYLIRPCEGGGSIIHIVDHLNLKAWTVPEVLRPLYESPNIIAQKMTIGALRYIR
QIAQETSGEIVYGLGRQPAVLRTFSQRLSRGFNDAINSFNDDGWSLLSSDGGEDVVIAINSSKNLASGSNNLSFI

GGVLCAKASMLLQNVPPAVLVRFLREHRSEWADFNVDAFSAASLKASPYAYPGMRPTRFTGSQIIMPLGHTIEH
EEMLEVIRLEGHSLGQEDAFISRDIHLLQMCSGIDENAVGACSELVFAPIDEMFPDDAPLVPSGFRIIPLESKPGES
QDTLTAQRTLDLTSSLEVGPETNHGGGKATTSSYGARSVLTIAFQFPFESNLQDNVASMARQYILSVISSVQRVA
MAISPSGLTPKLSPASPEALTLAHWICQSYSFHVGTELVRAEAIGGESMLKSLWHHQDAILCCSLKQALPVFIFAN
QAGLDMLETTLVALQDITLDKIFDDGGRKALFSEFAKIMQQGFAYLPGGMCMSTMGRHISYEQAIVWKVFAA
DETTLHCLAFSFVNWSFV
>c76574
VNSSPRQAPIPFNFTIFNSSTYCPQDVAKMVQSSALNYQMLIVTQLKWAWDYLLYNSFFNTRGIMIPAYDDDLS
VTRYERGAGNDENLEDCAVCLCKIDDGDAVRELNCNHLFHRVCLDRCIGYGHATCPLCRDNVIKPPRLRWMAT
KRLY
>AtWUS
MEPPQHQHHHHQADQESGNNNNNKSGSGGYTCRQTSTRWTPTTEQIKILKELYYNNAIRSPTADQIQKITARL
RQFGKIEGKNVFYWFQNHKARERQKKRFNGTNMTTPSSSPNSVMMAANDHYHPLLHHHHGVPMQRPANS
VNVKLNQDHHLYHHNKPYPSFNNGNLNHASSGTECGVVNASNGYMSSHVYGSMEQDCSMNYNNVGGGW
ANMDHHYSSAPYNFFDRAKPLFGLEGHQEEEECGGDAYLEHRRTLPLFPMHGEDHINGGSGAIWKYGQSEVR
PCASLELRLN

Nucleotide sequences

>JsWOX1
ATGTGGATGATGGGATACAACGATGGAGGAGGCGATTTCGACCCCACCGGCTGGAAATTCCGACCACTTGT
CCCCCGACCGGCCGCCACTTCCGCCACCGTTAACCCCCATTGCTTCAGCCGTGTAAACCCCACTGATCTTCTT
TCCCTAAATCACCACCTCTGTGTGGCAGAGAAGAACAAGAGAGAGTTCAACACGCAGCAGGTGGTGGCGA
GCTCGCGGTGGAACCCTACGCCGGAGCAACTGCAGACGCTGGAGGAGTTGTATCGACGGGGGACGAGAA
CACCGTCGGCTGATCAAATTCAGCACATTACTGCACAGCTTAGGCGTTATGGAAAGATTGAAGGGAAGAAT
GTGTTTTACTGGTTTCAAAATCATAAAGCTAGAGAGAGGCAAAAAAGGCGACGTCAACTGGAATCTGTCTCT
GATAGTGAACAACAACCTCGGAGTAACATGGAGATTTCCGAAAAGAAAGAATCTTCAGATGCAAACAGAAC
AATATATGAATGTGAACAGACTAAAAATTGGGCATCTCCTACAAACTGTAGTACAATTGCAGAGAAAACCCTA
CCATCAACACAAAAGACACCAGAAGCAGAATGTAAATCAGATGGATGGCTACAGTTCGATGAGGTTGCTCAT
GAATTACAACAAAGGAGGAGCCAGCTGGCAGAAATGAATGCCACGTGGCAAACCACCAAACAATCAATTA
ACCCCACTAATGACGTCATAATCACGTTCTTACCGGCAAGAAGAGACGACGAATTTCAGACCCTTCAACTCTT
TCCTCTCCAAAGCAGTGACGGCAGTGGTGGTGGTGATTGTGGCGACAACGATGATCATTCGCAGGAGAATG
TGGTGCAGACATCGGCTTCCGGCATGAATTCTGACCTCAGTTCTTATCAGTTTTTTGAGTTCCTTCCATTGAA
GAACTGA
>JsWOX4
ATGGTAGTTGAAGCCACCATGAAGGTTCATCAATTTGCACGTGGATTTTGGGATCAACATGTGCACGAGACA
CCCTCACTAACTCTCGGGTGCAAGCGCTTTCGTCCGCTCGCCCCCAAGCACCTCACGGCCAAAAACTCGGA
CACTAACGACCCCATTTTCGACCTCAAGAGCTTCATTAGACCTGAGAGTGGGCCCATCAAGATTGGATCGTC
CGATTATCACAAGAAAGAATCCGCTCAGGTGGAGACGCACCCTGGAGGGACGAGGTGGAACCCGACCCAA
GAACAGATAGGGATACTTGAAATGTTGTATAGAGGAGGAATGCGAACGCCAAACGCTCAACAAATCGAGCA
AATCACAGCTCAGTTAGGGAAGTATGGCAAGATTGAAGGCAAAAACGTGTTTTACTGGTTCCAAAACCACA
AAGCACGAGAGAGACAGAAGCAAAAGCGCAATAGTCTAGGTCTTAGCAGCCATAGCCCACGAACACCACC
TTCCATTCTCAACACTTCTCTATTATTATCTGAAAATAAGGGGGACGAAGACAGTCCGCATAAGAGAAAGTGC
AGGCCATGGACATTCGAAGGCTTCGACGAGGAGAAGAGATATTGTACAGAAGACGAAGACGAAGATAGGA

CTTTGAAACTCTTTCCCTTGCACCCCGAAGGACGATGA

>JsWOX13x1

ATGGAGTGGAATAAACCGCCGCCGCAGCAGGCGGCGCATCCGGAGGAACTGAACGGAGGAGGTGGTGGT
GGTGGAAATTTGAATGTCAACGGCGGAGGAATGTTTGTTAAAGTGATGACCGATGAGCAAATGGAAGTTCT
CCGAAAGCAGATTGCAGTATACGCCACCATTTGCGAACAGCTTGTCGAATTGCATAAATCGCTCACTTCTCAA
CACGATCTCGCCGGTGCCAGATTGGTAAATCTATATTCCGATCCTTTCGTAACATCTGGAGGTCATAAAATTAC
GGGCAGGCAGAGATGGACTCCAACACCAGTCCAACTTCAGATTCTTGAAAGAATATTTGATCAAGGAAATG
GAGCTCCAAGCAAACAGAAGATCAAAGAGATAACAGCAGAGTTATGCCAACACGGCCAGATTTCTGAGAC
AAATGTTTATAATTGGTTTCAGAATAGACGTGCTCGATCAAAGAGGAAGCAACAGGCTACACAACAAAACCA
TGCTGAATCAGAAGTGGAGACAGAGGTTGAGTCACCAAATGAAAAGAAAACTAAGCCTGATGATTTTCAAA
CTCCGCAAATCGCAAGTTCAAAGCCCGAAGATCTTTGCTTTCAGAACCCTGAGGCTAATTCTGCTATGCATTC
CATGGATCCACGTGCCAGTAAGCCTGAACCCATGTTGCCACCAGAAGGCAGCTCACATCACAGTGGGAATT
TCGGGCAGATGTCCTTCTATGGGAATATGTTAGTAAATCAGAGGATGGACAACATGATTGGAAAGATGGAAG
TTCCAGGAAACTATAATCCTTATCTGCAACCAGATGACTACAACATGACAAGGTAG

>JsWOX13x2

ATGGAGTGGGGAAAACAACCAAACGTGCAGCAAGTAGCAGAGGAACTTAATAATGGAGGAATTTTTGTGA
AGGTGATGACAGATGAACAAATTGAAATCCTCCGTAAGCAGATCGCTGCTTACGCCATCATTTGTGAGCAAC
TTGTCCATTTACACAAATCCTTCACTCTTCAAAACGATCTTCCAGGGTATGGAAATCTGTATTATGGCCCCTTG
ATGTACCCTGGTGGCCATAGAATTTCTGCCAGACAAAGGTGGACTCCAACACCAGTTCAACTTCAGATTCTT
GAGCGTACATTCGACCTAGGGAATGGAACTCCAAGCAAGCAGAGAATCAAAGAGATAACATCTGAATTGTC
TCAACATGGGCCAATTTCAGAGACTAACGTTTATAATTGGTTTCAAAACAGGCGTGCCCGATCAAAACGGAA
GCAACAATCTGCTCCACTGTATTCTGCTGAACCAGAAGCAGACACAGATGTTGAGTCTCAAAGTGAGAAGA
CCGAAAAGCCTCAGCAGCACGTCCCAAGTTCAAGGTCTGAAGATAACCCCGAGTTTAGTTCTGCAACACAC
TCCATGGATCCATTCAGAATGAGGAAACCTGAAAGGATTTTGCAGCCAGAAGATAGTTTGAAGCGTAATGG
GAGTTCTGGTCAACTGCCATATGGAAGCATGTTATCAAATCCTGGAGTGGAGCACTTAATTGAGAAGATGGA
AGTTCCGGGGAGCTATAGTTCTTACCCACAATTAGATGGCTTCGACCCAACTGGCTAA

> c19299

GAGTCGGAAAACATGAACTGGATATTGGCCAACTCCAAGCCTTGTCCCAAATGCAAGCGACCAATCGAAAA
GCATCAAGGTTGTATGCACATGACTTGCACGCCACCCTGCAAATTTGAGTTTTGCTGGCTGTGTCTAGGTGC
ATGGTCAGAGCATGGTGAGAGGACTGGAGGTTTCTATGCATGCAACCGATACGAGACAGCAAAGCAAGAG
GGAGTGTACGATGAAGCGGATAGGAGGAGAGAGATGGCCAAGAACTCTTTGGAACGATACACTCATTACTA
CGAGCGATGGGCTTCCAACCAATCTTCAAGGCATAAGGCGTTAGTGGATCTGCATCAAATGCAAAGTGTTCA
TCTCGAGAAGCTTAGTGAAGTACAATCCCAACCGGAGTCACAAATGAAGTTCATTATAGAATCCTGGCAGCA
AATTGTTGAGTGTAGGAGAGTGTTAAAATGGACCTATGCTTATGGATACTACCTTCCTGAACATGAGCATACA
AAGAAGCAGTTCTTTTAG

> c92402

ATGCCTGCAATGGAGTCAATAGAAGCTCCGGCTAACGATGATGGGATGGCAGGGTTTCTCCCACTCGCTTCC
GCTTCTCAGCAGCCCTACGTCTCTGAACTGCTTTCATTTACTCTCGATCGCCTCCACAAGGAGCCAGAGCTAT
TGAGGGTAGATGCGGAGAGAATTCAGAGGCAGATGCAGGAGGTGGCCGTAGGAAATTTTCGAGCGTTCAT
TGCAGCGGCCGATGCCTTGCTCGAGATCCGAGAGGAGGTGTCCTCTATCGATAGACACCTCGAATCTCTGAT
AGCTGAAATCCCCAAGCTAACTTCTGGTTGCAGTGAGTTTCTTGAGTCAGCAGAGCAAATTTTGGAGAAGA
GGAAGATGAATCAAACCTTGCTCACCAATAGTAGTACATTGCTTGACTTGCTTGAAATTCCTCAGCTTATGGA
CACATGTGTGCGAAATGGAAATTATGATGAAGCTCTTGACTTGGAAGCATTTGTCGTCAAACTTACAACAAT
GCATCCAAAAAATTCCTGTGATTCAGGATCTTGCTGCAGAAGTTAAGCAGACCACTCAGTCTCTTCTTTCTCAG

CTTCTCCTAAAACTTAAATCAAACATTCAGTTGCCAGAGTGCCTTCGCATCATTGGGTATTTACGTCGAATTGG
AGTCTTTAGCGAGTATGAAATGCGCCTACAGTTTTTGAGATGTCGTCAAGCATGGCTTACCGGAATACTTGAT
GACTTAGACCAGAGAAATCCATATGAATATTTAAAAGGGATGATAAATTGTCACAGAATGCATCTTTTTGATG
TTGTTAACCAATACCGAGCCATATTTGCGGATGATACATCGGGAATAGAAGAAAATCATGATGGGGGGCTTC
TCTTCAACTGGGCCTTGCATCAAATCACGTCTCACTTGAAAACTCTCAAAGTCATGCTCCCTAAGATAAGTGA
AGGAGGGTCTTTGTCAAATATTCTAGATCAATGCATGTATTGTGCCATGAGTCTTGGCTGGGTTGGCTTGGAT
TTTCGCAGCTTGCTTCCACCTCTATTTGAAGAAGCAGTCGTTAACTTATTCTCAAAGAATATGAGCACAGCTG
TTGAAAATTTTCAGTTAGTGTTGGATTCTCATCGTTGGGTCCCGTTACCAGCAGTTGGCTTTCCAGCCAATAG
TTTGGGTGAAGAAAATCAGGATGATGTTACTCCTCCCTCTAATCTTATGGAGCATCCACCTCTTGCTGTTTTTA
TAAATGGTGTATCAGCTGCGATGAATGAATTACGTCCTTGTGCTCCATTAAGTTTGAAAAACTTGCTTGCTCA
AGAGTTAGTCAAGGGACTGCAAGCTGTTTCTGATTCTTTATTGAGATACAAAACAACTAGGATGCTCAGAGA
TAACGAATCATTACTTTTTCTTAAGCTTTGCCATGCATTTATCGAGGTTGCCTTTCCACATTGCGTCACCTGCTT
TGGACGCTGTTACCCCGGTGGAGCTTCTCTGATTGCAGATGCCAAGAATTTGTTTGATGGAATCAATCGCTTA
CTGGAGAACTCTCGCTCAAGAGCACTACCTAAACAAGTACACCGTGCAGACTCGAAGAGCACGTCAGAGA
ACGGGAACCAACCCGTGGCAGAGAATGGGAATCAACCCGTGGCAGAGAATGGGATTGTACATTCAGATGA
ACAAGCAGCAAGTGACAATGATAGGGAAAAAGAACATAATAATATCAGCCCACAAAGGAGGAGCTCAGTAA
CAGATGAAGATAATTCAGATCCGCTGCCTCAAGAAGTGAAGACATAG
> c16725
ATGGCTATGGTAGTACAGCAACACAGGGAGAGTAGCAGCGGCAGCGGCAGTGGCGGTGGGAGCATAAAC
AAACATCTTGACACCGGAAAATATGTCAGGTACACTGCGGAGCAGGTGGAGGCGTTGGAGAGGGTATATTC
CGAGTGCCCAAAACCCAGTTCTTTGCGCCGCCAACAACTCATCAGAGAATGCCCTATTCTTTCCAACATTGA
ACCCAAACAGATTAAAGTCTGGTTCCAAAATCGCAGATGTCGGGAGAAGCAGAGGAAAGAATCTTCTAGAC
TTCATAGTGTGAACAAGAAATTGAGTGCGATGAACAAGCTGTTGATGGAGGAAAACGACCGCCTTCAGAAG
CAGGTTTCACAGCTCGTCTGCGAGAATGGCTATATGCGTCACCAATTGCAAAGTGCAACAACTGATGCGAGT
GGTGAATCTGCATCGACCACTCCTCGTAATGCTATCAGAGATGCTAACAATCCTGCTGGACTCCTCTCTATCGC
GGAGGAGACTTTGGCAGAATTCCTATCAAAGGCCACAGGTACTGCTGTCGATTGGGTCCAGATGCCTGGGA
TGAAGCCTGGTCCGGATTCAGTTGGGATCTTTGCCATTTCACAAAGTTGTGGTGGAGTGGCAGCTCGAGCC
TGCAGTCTTGTTAGTTTAGAACCTACAAAGATTGCAGAGATCCTTAAAGATCGATCTTCTTGGTTTCGGGACT
GTCGAAGTGTCGAAGTTTTCACTATGTTTCCTGCTGGAAATGGAGGAACTATTGAGCTTTTGTATACACAGA
CGTATGCACCGACTACACTGGCTCCTGCTCGTGACTTTTGGACTCTGCGATACACGACAACCTTAGAAAACG
GAAGTCTCGTGGTGTGTGAAAGATCTCTTTCAGGTACCGGGGCAGGCCCAAGTGCGGCATCAGCTTCACAG
TTTTTAAGGGCTGAGATGCTTCCTTCTGGATATTTGATCCGACCGTGTGAGGGTGGAGGTTCAATTATTCATA
TCGTCGATCACCTCAATCTCAAGGCCTGGACTGTGCCGGAGGTGCTTCGCCCACTTTATGAATCACCCAATAT
TATAGCACAGAAAATGACTATTGGAGCACTGCGATATATCAGGCAGATAGCTCAGGAGACGAGTGGTGAGAT
AGTATATGGTCTTGGTCGGCAACCAGCTGTTCTACGCACTTTTAGCCAGAGATTAAGCAGAGGTTTTAATGAT
GCTATTAACAGCTTCAATGATGACGGCTGGTCGCTATTGAGCTCCGACGGGGGTGAAGATGTAGTAATTGCT
ATCAACTCCTCGAAAAACCTTGCGAGTGGATCAAATAACCTTTCGTTTATTGGTGGCGTCCTTTGTGCCAAAG
CTTCTATGCTACTTCAGAACGTGCCTCCAGCAGTACTGGTTCGGTTTCTGAGGGAGCATCGTTCAGAGTGGG
CCGACTTTAATGTGGATGCCTTTTCTGCTGCTTCTTTGAAAGCAAGCCCATATGCCTATCCGGGAATGAGGCC
CACGAGGTTTACTGGGAGCCAAATCATCATGCCACTAGGTCATACTATTGAACATGAAGAAATGCTTGAAGT
GATTAGGCTGGAGGGACACTCTCTAGGTCAAGAAGATGCTTTCATCTCGAGGGACATTCACCTCTTACAGAT
GTGTAGCGGGATTGATGAAAATGCAGTGGGAGCCTGCTCCGAGCTGGTTTTTGCTCCGATCGACGAGATGT
TTCCGGATGATGCACCATTAGTGCCGTCTGGTTTCCGCATAATCCCCCTCGAATCAAAACCAGGGGAGTCAC
AGGATACATTGACGGCGCAAAGAACGCTAGATTTGACATCTAGCCTCGAAGTCGGCCCAGAAACGAACCAT

GGAGGTGGTAAGGCAACAACGTCGTCTTATGGTGCACGATCAGTATTGACTATTGCATTCCAATTCCCGTTTG
AGAGCAATTTACAGGACAATGTTGCATCAATGGCTCGTCAGTACATTCTTAGTGTGATTTCCTCGGTGCAAAG
GGTTGCAATGGCAATATCTCCGTCGGGATTGACTCCTAAGCTATCCCCAGCCTCTCCAGAAGCCCTTACTCTA
GCTCATTGGATCTGTCAAAGCTATAGTTTCCATGTGGGAACCGAATTGGTGAGGGCTGAAGCCATTGGCGGT
GAATCGATGTTGAAATCTCTGTGGCATCACCAAGATGCCATATTGTGCTGCTCTTTGAAGCAAGCGCTGCCA
GTTTTCATATTCGCTAACCAGGCTGGGCTTGACATGCTGGAGACGACTCTGGTGGCTTTACAAGACATTACAT
TAGATAAGATATTTGACGATGGTGGTCGCAAGGCACTCTTCTCTGAATTCGCTAAGATAATGCAACAGGGATT
TGCTTACTTGCCGGGTGGCATGTGCATGTCGACAATGGGGCGCCATATCTCTTACGAACAAGCAATTGTGTG
GAAAGTGTTTGCAGCGGACGAGACGACCCTCCACTGCCTCGCCTTCTCTTTCGTGAACTGGTCGTTCGTCTA
A

> c76574

GTCAATTCAAGCCCTAGACAGGCCCCTATTCCATTTAACTTCACAATCTTCAACAGCAGTACATATTGCCCACA
AGATGTTGCCAAAATGGTGCAATCAAGTGCATTAAACTATCAAATGCTGATTGTCACTCAGCTCAAGTGGGC
ATGGGATTATCTTCTCTATAACTCCTTCTTCAACACTCGTGGAATCATGATACCCGCATATGACGATGATCTTAG
CGTAACACGATACGAGAGAGGTGCGGGAAATGATGAGAATCTAGAGGACTGTGCTGTGTGTTTATGCAAGA
TTGATGATGGAGATGCAGTTAGGGAGTTGAATTGTAATCATCTTTTCCACAGAGTTTGTTTGGATAGGTGTAT
TGGATATGGGCACGCCACGTGTCCTCTGTGCCGGGATAATGTTATTAAGCCGCCTCGACTGCGGTGGATGGC
CACCAAGAGGTTATATTGA
