# Supplementary figures and images for: Characterization of JsWOX1 and JsWOX4 during Callus and Root Induction in the Shrub Species Jasminum sambac
Source: Plants (Basel). 2019 Mar 29;8(4):79. doi: 10.3390/plants8040079 (PMC6526479; doi:10.3390/plants8040079)

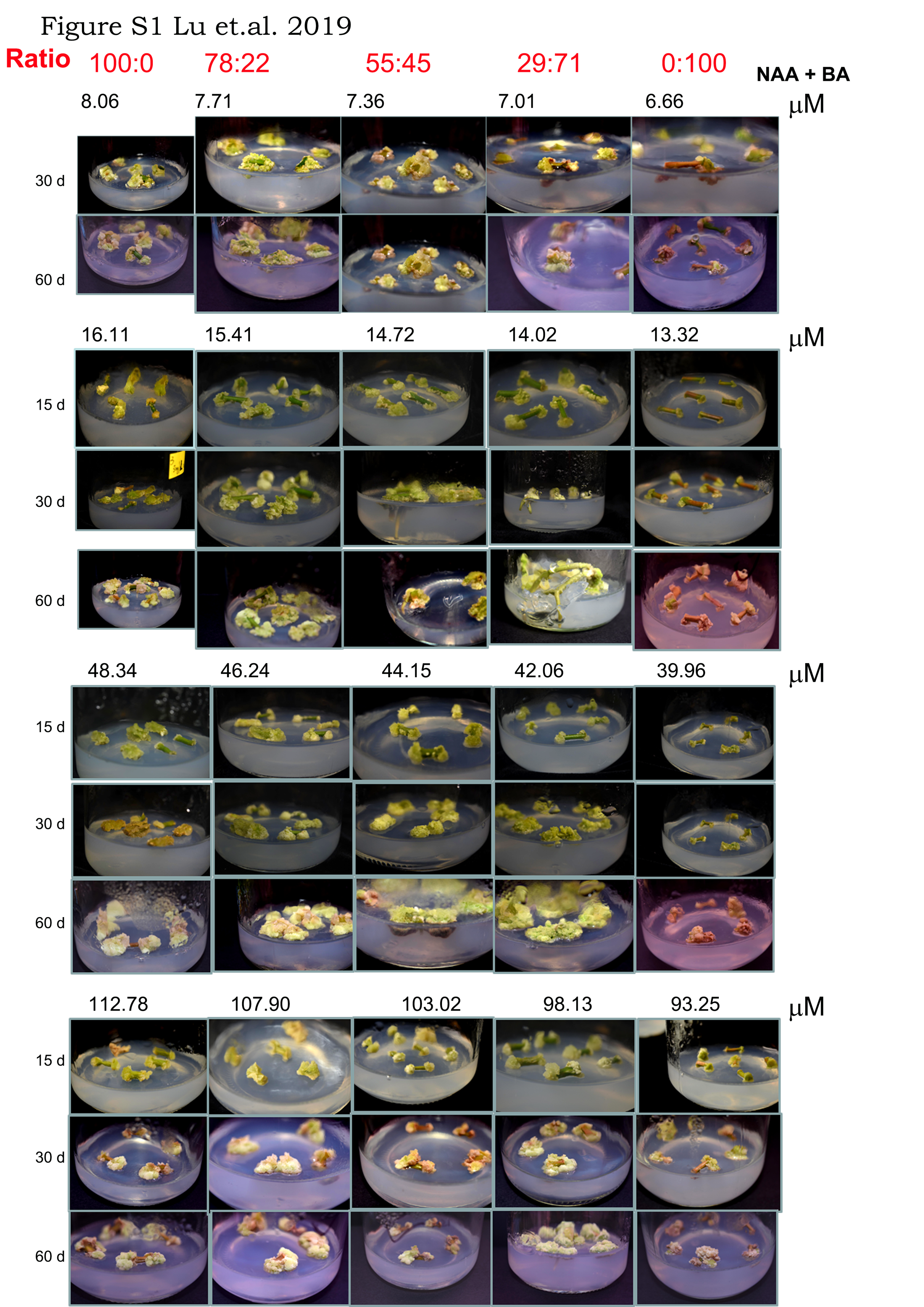

Supplement: Supplementary file 1 [file plants-08-00079-s001.zip › Figure S1.tif]
